# From Sea to Relief: The Therapeutic Potential of Marine Algal Antioxidants in Pain Alleviation

**DOI:** 10.3390/md23070270

**Published:** 2025-06-27

**Authors:** Mariola Belda-Antolí, Francisco A. Ros Bernal, Juan Vicente-Mampel

**Affiliations:** 1 School of Medicine and Health Science, Department of Physiotherapy, Catholic University of Valencia, 46002 Valencia, Spain; mariola.belda@ucv.es; 2 Predepartamental Unit of Medicine, School of Health Sciences, Universitat Jaume I, 12006 Castellón de la Plana, Spain; fros@uji.es

**Keywords:** algae, bioactive compounds, antioxidants activity, chronic pain, pain management

## Abstract

Chronic pain affects approximately 20% of the global adult population, posing significant healthcare and economic challenges. Effective management requires addressing both biological and psychosocial factors, with emerging therapies such as antioxidants and marine algae offering promising new treatment avenues. Marine algae synthesize bioactive compounds, including polyphenols, carotenoids, and sulfated polysaccharides, which modulate oxidative stress, inflammation, and neuroimmune signaling pathways implicated in pain. Both preclinical and clinical studies support their potential application in treating inflammatory, neuropathic, muscular, and chronic pain conditions. Notable constituents include polyphenols, carotenoids (such as fucoxanthin), vitamins, minerals, and sulfated polysaccharides. These compounds modulate oxidative stress and inflammatory pathways, particularly by reducing reactive oxygen species (ROS) and downregulating cytokines such as tumor necrosis factor-alpha (TNF-α) and interleukin-6 (IL-6). Brown and red algae produce phlorotannins and fucoidans that alleviate pain and inflammation in preclinical models. Carotenoids like fucoxanthin demonstrate neuroprotective effects by influencing autophagy and inflammatory gene expression. Algal-derived vitamins (C and E) and minerals (magnesium, selenium, and zinc) contribute to immune regulation and pain modulation. Additionally, sulfated polysaccharides suppress microglial activation in the central nervous system (CNS). Marine algae represent a promising natural source of bioactive compounds with potential applications in pain management. Although current evidence, primarily derived from preclinical studies, indicates beneficial effects in various pain models, further research is necessary to confirm their efficacy, safety, and mechanisms in human populations. These findings advocate for the continued exploration of marine algae as complementary agents in future therapeutic strategies.

## 1. Introduction

Pain is a multifaceted physiological phenomenon initiated by the activation of nociceptors, which are sensory neurons that respond to actual or potential tissue damage. Nociception is categorized into four stages. The first stage, transduction, involves the conversion of noxious stimuli into electrical impulses by primary afferent neurons. The second stage, transmission, refers to the propagation of these impulses to the central nervous system (CNS) via A-delta and C fibers [1]. The third stage, called modulation, occurs in the spinal cord and brainstem. Here, pain signals are controlled by a mix of chemicals. Some chemicals, such as glutamate and substance P, increase the signals. Others decrease signals, such as GABA and serotonin [2]. Finally, perception, the conscious experience of pain, arises from the processing of these modulations within higher cortical structures, integrating sensory, cognitive, and emotional dimensions [3].

Beyond this fundamental nociceptive pathway, pain can be broadly classified based on its duration into acute or chronic pain, as well as by its underlying mechanisms [4]. Traditionally, pain is classified into two types: nociceptive and neuropathic [3]. Nociceptive pain occurs when nociceptors are activated. This activation releases chemicals that make pain pathways more sensitive to pain. However, neuropathic pain is different. It involves complex changes in the nervous system. These changes include unusual nerve signals, changes in nerve cell genes, problems with ion channels, and abnormal immune system activity [5]. Pain processing requires a careful balance between pathways that increase and decrease signals in both the peripheral and central nervous systems [6]. Persistent pain can cause significant changes in the brain and spinal cord.

These changes can make a person more sensitive to pain, known as nociplastic pain [7]. This prolongs pain and makes it more complex. To manage pain effectively, we need to focus on identifying and treating the real causes of pain, not just the symptoms [8]. Understanding these causes is important for developing better methods for diagnosing and treating different types of pain [9]. Although pain can be managed in many ways, such as through medication or therapy, these often have side effects that can harm patients and reduce their effectiveness [10]. According to the International Association for the Study of Pain, approximately 20% of the adult population worldwide suffers from chronic pain, with a substantial proportion experiencing moderate-to-severe pain [11].

Direct healthcare costs, such as doctor visits, treatments, and medicines, place a significant strain on healthcare systems [12]. However, indirect costs, such as lost work time, missing work, and expenses related to disability, are often much higher [13]. A study published in the journal *Pain* found that chronic pain costs in the United States are between USD 560 billion and USD 635 billion each year [14]. Recognizing this profound impact, the World Health Organization (WHO) has recognized the profound impact of chronic pain on mental health and overall well-being [15]. This emphasizes the need for comprehensive pain management strategies that address both the physical and psychosocial dimensions of this complex condition [16].

The pathophysiology of chronic pain is frequently associated with persistent inflammation and increased oxidative stress. A key mechanism contributing to this process is the generation of reactive oxygen species (ROS), which induce cellular damage and sensitize nociceptors, thereby exacerbating pain perception. Conversely, the administration of antioxidants, which mitigate oxidative stress and dampen inflammatory responses, presents a potential therapeutic avenue for reducing pain intensity [17]. In summary, effective management of chronic pain remains a substantial clinical challenge. This highlights the need for new and personalized treatments. In this context, the discovery of pain-relieving properties in marine algae offers new opportunities for developing innovative treatments.

### 1.1. Oxidative Stress and Marine Algae

Oxidative stress is a critical etiological factor in numerous inflammatory and neurodegenerative diseases associated with chronic pain [18]. Overproduction of ROS can damage cells and tissues, thereby exacerbating inflammation and pain perception [19]. In this context, natural antioxidants have emerged as promising therapeutic agents.

Marine algae have been employed for centuries in traditional medicine across diverse cultures, particularly in Asia, owing to their remarkable nutritional and therapeutic properties. Contemporary science has substantiated many of these traditional medicinal applications, demonstrating their efficacy in neutralizing free radicals and shielding cells from oxidative damage [20]. These marine organisms represent one of the richest natural sources of antioxidants and other bioactive compounds capable of modulating inflammatory and nociceptive pathways [21].

Recent investigations have explored the impact of antioxidant compounds derived from algae on pain management, particularly in inflammatory conditions such as arthritis, autoimmune diseases, and musculoskeletal disorders [22,23]. Certain marine metabolites can influence the regulation of pro-inflammatory cytokines and diminish pain sensitivity through mechanisms involving the modulation of oxidative stress and inflammation [24]. Notably, studies in experimental models have shown that compounds such as fucoidans extracted from brown algae can suppress the expression of inflammatory mediators such as tumor necrosis factor-alpha (TNF-α) and interleukin-6 (IL-6), which are implicated in the amplification of inflammatory pain [25,26,27].

Furthermore, it has been observed that carotenoids, such as astaxanthin, present in some species of red algae, can exert analgesic effects by modulating signaling pathways related to oxidative stress and microglial activation in the CNS [28,29,30]. Similarly, polysaccharides, such as laminarins, have been investigated for their capacity to mitigate inflammation in preclinical models of neuropathic pain [31]. This review critically assesses the existing scientific literature on antioxidant compounds derived from marine algae and their demonstrated or potential applications in pain management. Additionally, it evaluates the evidence supporting their efficacy and explores future opportunities for their integration into pharmaceutical and nutraceutical formulations targeting both chronic and acute pain conditions.

#### Antioxidant Properties of Algae

Algae exhibit remarkable diversity, encompassing 4 kingdoms, 14 phyla, and 63 classes [32]. The major groups include blue-green algae (*Cyanophyta*), green algae (*Chlorophyta*), red algae (*Rhodophyta*), and various chromists, such as diatoms and dinoflagellates [32,33]. This diversity stems from the different evolutionary histories of plastids, primarily derived from endosymbiotic events involving cyanobacteria [34,35]. Algae are classified based on their photosynthetic pigments, cell structures, and biochemical characteristics [33]. The most species-rich phylum is Heterokontophyta, which is dominated by diatoms, followed by red algae, green algae, and cyanobacteria [32]. Algae play crucial roles in aquatic ecosystems and have significant economic importance, with potential applications in biotechnology [34]. Their diverse photosynthetic mechanisms and pigments continue to be rich sources for scientific exploration and industrial innovation. This diversity arises from the distinct evolutionary trajectories of plastids, primarily through endosymbiotic events involving cyanobacteria. Algae synthesize a diverse array of bioactive compounds that exhibit antioxidant properties. These compounds include polyphenols, carotenoids, vitamins, minerals, and sulfated polysaccharides, which are particularly prevalent in marine algal species.

### 1.2. Polyphenols: A Rationale for Their Investigation

**Figure 1:**
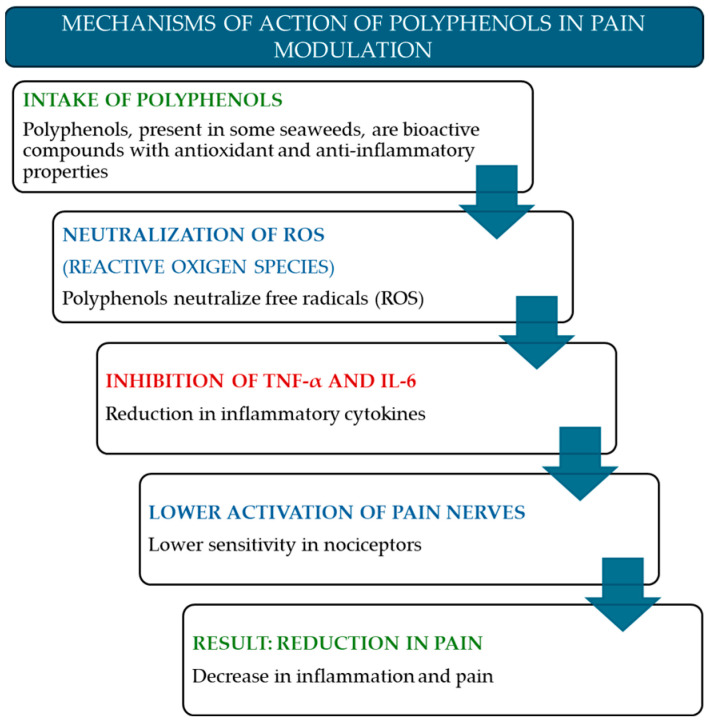
Step-by-step process by which polyphenols—bioactive compounds found in certain seaweeds—modulate pain. The pathway includes their antioxidant effects through the neutralization of reactive oxygen species (ROS), inhibition of pro-inflammatory cytokines (TNF-α and IL-6), reduced activation of pain-related neurons, and the outcome of decreased inflammation and pain perception.

Marine polyphenols, a diverse class of secondary metabolites found in various algal species, have emerged as promising candidates for developing novel anti-inflammatory and analgesic therapeutic strategies. These bioactive compounds are instrumental in modulating key pathways involved in inflammation and oxidative stress, thereby contributing to pain reduction in a wide range of musculoskeletal and joint disorders (Figure 1). This subsection presents a rational argument for the potential of these compounds, drawing upon existing evidence to elucidate their mechanisms of action and highlight their therapeutic relevance (Table 1).

Brown algae, including *Fucus vesiculosus*, *Ecklonia cava*, and *Ascophyllum nodosum*, are rich sources of polyphenolic compounds exhibiting significant anti-inflammatory and analgesic effects [36,37,38,39]. Mechanistically, these polyphenols inhibit the production of pro-inflammatory cytokines, notably TNF-α and IL-6, thereby directly addressing a critical step in the inflammatory cascade. This cytokine inhibition leads to demonstrable mitigation of inflammation and a subsequent reduction in perceived pain. Furthermore, the inherent antioxidant properties of these compounds contribute to tissue protection by scavenging ROS and reducing oxidative damage. This dual action likely facilitates accelerated recovery from musculoskeletal disorders [40,41].

Notably, recent studies have indicated that phlorotannins, a specific class of polyphenols prevalent in brown algae, can modulate the activity of matrix metalloproteinases (MMPs). Given the established roles of MMPs in cartilage degradation in conditions such as rheumatoid arthritis, this finding suggests a potential chondroprotective role for these compounds in degenerative joint diseases [42]. Similarly, red and brown algae species such *as Laminaria japonica*, *Sargassum muticum*, and *Undaria pinnatifida* are recognized for their polyphenol content and associated anti-inflammatory and antioxidant properties. *Laminaria japonica* is particularly rich in phlorotannins, which have been shown to effectively mitigate oxidative stress and decrease the production of key inflammatory mediators, including interleukin-1 beta (IL-1β) and prostaglandin E2 (PGE2). This modulation of inflammatory signaling pathways provides a plausible mechanism for the observed pain-alleviating effect. *Sargassum muticum* also contains phlorotannins, which have been shown to exert regulatory effects on the nuclear factor kappa-light-chain-enhancer of activated B cells (NF-κB) pathway, a central regulator of inflammatory responses. This regulation results in a downstream reduction in pro-inflammatory mediators. In *Undaria pinnatifida*, the presence of both fucoxanthin and phlorotannins contributes to its bioactivity. The potent antioxidant properties of these compounds, specifically their ability to inhibit the formation of ROS, play a crucial role in modulating the inflammatory response and protecting against cellular damage [39,43,44]. Recent research has increasingly focused on the therapeutic potential of marine algal polyphenols in inflammatory and degenerative diseases, including arthritis and fibromyalgia. The convergence of their antioxidant properties and their capacity to modulate key inflammatory pathways positions them as promising natural agents for effective pain management. Further rigorous investigation, including well-designed preclinical and clinical studies, is warranted to fully elucidate their therapeutic potential and pave the way for their integration into evidence-based treatment strategies for these debilitating conditions.

### 1.3. Carotenoids: A Rationale for Their Bioactive Potential

Fucoxanthin is a distinctive carotenoid widely distributed in brown algal species, where it plays essential roles in photosynthetic processes and provides cellular defense against oxidative stress. It has garnered significant scientific interest due to its reported antioxidant, anti-inflammatory, and neuroprotective properties. This subsection presents a focused analysis of the potential therapeutic applications of fucoxanthin, drawing on existing evidence to elucidate its mechanisms of action across these domains (Table 2).

Fucoxanthin is found in various brown algal species, *including Undaria pinnatifida*, *Laminaria japonica*, *Sargassum horneri*, *Fucus vesiculosus*, *Ascophyllum nodosum*, and *Hijikia fusiformis*, and has demonstrated a diverse array of beneficial biological effects. Notably, its significant antioxidant and anti-inflammatory properties suggest a potential role in mitigating inflammation-induced hyperalgesia in the present study. Mechanistically, fucoxanthin exerts its effects on inflammatory signaling pathways by downregulating the expression of pro-inflammatory genes, including TNF-α, IL-6, and NF-κB [45]. Furthermore, it alleviates pain by reducing the production of ROS, thereby mitigating cellular damage and preserving the functionality of tissues affected by chronic inflammation [46,47,48]. For instance, studies on *Sargassum horneri*, have demonstrated that fucoxanthin effectively suppresses the production of pro-inflammatory cytokines by microglial cells, consequently safeguarding cells against oxidative stress [47,49].

Emerging research has also highlighted the neuroprotective potential of fucoxanthin. Evidence suggests that it can modulate autophagy in neuronal cells, opening novel avenues for its application in neuroprotection. This mechanism is particularly relevant in the context of neurodegenerative diseases, such as Alzheimer’s and Parkinson’s diseases, where neuronal survival and reduction in neuroinflammation are critical therapeutic targets [50,51]. In *Undaria pinnatifida*, fucoxanthin has been identified as a neuroprotective agent, as it reduces lipid peroxidation and enhances antioxidant function, indicating its therapeutic potential in preventing neuronal damage associated with neurodegenerative conditions [52]. The concentration of fucoxanthin varies across different algal species, with *Undaria pinnatifida* and *Laminaria japonica* containing up to 1.5 mg/g of dry algae [53]. Beyond its established anti-inflammatory and antioxidant properties, fucoxanthin derived from *Fucus vesiculosus* and *Ascophyllum nodosum* has demonstrated advantageous effects in the regulation of cytokines and modulation of oxidative stress [54]. These findings underscore the potential utility of curcumin in the treatment of metabolic and chronic diseases, where inflammation and oxidative imbalance play significant roles. In conclusion, fucoxanthin, a bioactive carotenoid found in various brown algae, exhibits compelling antioxidant, anti-inflammatory, and neuroprotective properties. These attributes suggest its potential as a therapeutic agent for managing pain, inflammation, and neurodegenerative diseases, such as Alzheimer’s and Parkinson’s diseases [51]. Further rigorous scientific investigations are warranted to fully elucidate its therapeutic efficacy and explore its potential for clinical translation.

**Table 2:** Quantification and mechanisms of action of fucoxanthin in algae.

Algae	Carotenoid	Quantification	Mechanisms of Action	Reference
*Undaria pinnatifida*	Fucoxanthin	0.2–4.5 mg/g DW	- Antioxidant: reduces ROS - Anti-inflammatory: inhibits expression of pro-inflammatory genes (TNF-α, IL-6, NF-κB) - Neuroprotective: modulates autophagy, improving neuronal function in neurodegenerative diseases (Alzheimer’s and Parkinson’s)	[52,53,54]
*Laminaria japonica*	Fucoxanthin	0.5–2.5 mg/g DW	- Inhibits lipid peroxidation - Potent antioxidant, protects against cellular damage in the CNS - Regulates the inflammatory response and modulates NF-κB signaling pathways	[53,54]
*Sargassum horneri*	Fucoxanthin	0.1–1.2 mg/g DW	- Anti-inflammatory: reduces the production of pro-inflammatory cytokines - Improves antioxidant function, protecting cells from oxidative stress	[47,53]
*Fucus vesiculosus*	Fucoxanthin	0.2–0.6 mg/g DW	- Inhibition of reactive oxygen species production - Modulates antioxidant pathways - Potential for treating chronic inflammation and metabolic diseases	[52,53]
*Ascophyllum nodosum*	Fucoxanthin	0.1–0.5 mg/g DW	- Prevents inflammation through regulation of cytokines - Acts as a modulator of oxidative stress	[53]
*Hijikia fusiformis*	Fucoxanthin	0.2–0.8 mg/g DW	- Neuroprotective effects by reducing neuronal oxidative stress - Potential for the treatment of Alzheimer’s disease and cellular protection	[53,54]

### 1.4. Vitamins and Minerals: Nutritional Significance in the Context of Inflammation

Various algal species, including *Porphyra umbilicalis*, *Ulva lactuca*, and *Gracilaria vermiculophylla*, are significant dietary sources of essential micronutrients, including vitamin C, vitamin E, selenium, magnesium, and zinc [55]. These compounds are critical for maintaining physiological homeostasis by mitigating oxidative damage and modulating the immune response, thereby bolstering the body’s inherent defenses against inflammatory and chronic disease states. This subsection presents a rational argument for the significance of these algal-derived micronutrients, detailing their established mechanisms of action (Table 3).

Vitamins C (ascorbic acid) and E (tocopherols) function as potent antioxidants, scavenging and neutralizing ROS, thereby safeguarding cellular components from oxidative damage. Specifically, in *Ulva lactuca*, the presence of vitamin C has been correlated with enhanced cellular function and tissue protection [56]. Concurrently, vitamin E contributes to the maintenance of membrane integrity and has demonstrated efficacy in mitigating oxidative stress associated with chronic inflammatory conditions [57,58]. Selenium, a notable compound of *Porphyra umbilicalis,* plays a crucial role as an antioxidant by enhancing the activity of endogenous antioxidant enzymes and bolstering the body’s capacity to neutralize damaging free radicals, thus mitigating cellular damage. Magnesium, which is abundant in *Gracilaria vermiculophylla*, has been implicated in the modulation of neuropathic pain. Its mechanism of action involves influencing the activity of N-methyl-D-aspartate (NMDA) receptors within the nervous system, suggesting its potential therapeutic relevance in the management of chronic pain [59]. Emerging evidence suggests that adequate magnesium levels may contribute to a reduction in overall pain sensitivity and alleviate conditions such as fibromyalgia and various forms of neuropathic pain. Furthermore, zinc, which is found across a range of algal species, is an indispensable trace element for optimal immune function. It plays a critical role in facilitating cellular repair and modulating inflammatory responses. Zinc has been demonstrated to enhance the performance of the immune system and serves as an essential cofactor for several key antioxidant enzymes which are crucial in protecting cells from the damaging effects associated with chronic inflammation.

In conclusion, the combination of vitamins C and E, along with essential minerals such as selenium, magnesium, and zinc, which are bioavailable from algal species, including *Porphyra umbilicalis*, *Ulva lactuca*, and *Gracilaria vermiculophylla*, represents a suite of crucial micronutrients. Their collective action not only mitigates oxidative damage but also plays a significant role in regulating immune responses, modulating pain pathways, and enhancing cellular protection against the development and progression of inflammatory and chronic diseases. These findings underscore the potential of incorporating algal-derived micronutrients as a rational component of dietary strategies aimed at promoting health and mitigating disease risks.

### 1.5. Sulfated Polysaccharides: Bioactive Agents from Marine Algae

Sulfated polysaccharides, a class of complex carbohydrates abundant in various marine algae species, particularly red and brown algae, have garnered considerable attention in the scientific community owing to their diverse pharmacological properties, notably their anti-inflammatory and neuroprotective effects. A growing body of evidence supports their capacity to modulate the immune system and reduce microglial activation within the CNS, suggesting a positive influence on the therapeutic management of neuroinflammatory diseases, such as fibromyalgia and multiple sclerosis [60,61]. This review presents a rational argument for the therapeutic potential of these compounds, focusing on the mechanistic insights that underpin their bioactivity (Table 4).

Recent studies have shown that specific sulfated polysaccharides, including fucoidan and carrageenan, can modulate the expression of genes intricately associated with the innate immune response. This regulatory function at the genetic level could be pivotal in conferring neuroprotection and in the broader management of autoimmune disorders, in which aberrant immune activity contributes significantly to disease pathology. These findings collectively suggest promising avenues for the development of adjunctive therapeutic strategies that specifically target inflammatory and neurodegenerative diseases associated with chronic pain syndromes [62]

Fucoidan and carrageenan sulfates, which are extracted from red and brown algae such as *Chondrus crispus*, *Kappaphycus alvarezii*, and *Sargassum wightii*, have consistently demonstrated both anti-inflammatory and neuroprotective properties in preclinical studies. Mechanistically, these compounds have been shown to influence the activity of the immune system, leading to measurable mitigation of microglial activation within the CNS. Given the established role of activated microglia in exacerbating neuroinflammation and contributing to altered pain processing in conditions such as fibromyalgia and multiple sclerosis, this inhibitory effect is of significant therapeutic interest [63,64]. Furthermore, emerging research suggests that fucoidan may exert its neuroprotective effects, at least in part, by modulating the expression of genes involved in the innate immune response, thereby offering a potential mechanism for neuronal protection in autoimmune disorders [65].

**Table 4:** Quantification and mechanisms of action of sulfated polysaccharides in algae.

Algae	Sulfated Polysaccharide	Quantification	Mechanism of Action	Reference
*Chondrus crispus*	Carrageenans	200–300 mg/g DW	Modulation of the immune system and reduction in neuronal inflammation	[65]
*Kappaphycus alvarezii*	Carrageenans	300–400 mg/g DW	Decrease in microglial activation and neuroprotection	[65]
*Sargassum wightii*	Fucoidan	40–80 mg/g DW	Regulation of immune response genes and antioxidant effect	[65]
*Undaria pinnatifida*	Fucoidan	20–80 mg/g DW	Reduction in pro-inflammatory cytokines and cellular protection	[65]
*Ecklonia cava*	Fucoidan	50–100 mg/g DW	Activation of anti-inflammatory pathways and neuroprotection	[65]

In conclusion, accumulating evidence strongly supports the rationale for exploring sulfated polysaccharides from marine algae as potential therapeutic agents for neuroinflammatory and autoimmune conditions characterized by chronic pain. Their ability to modulate immune responses and protect the nervous system through distinct mechanisms warrants further rigorous investigation, including comprehensive preclinical and clinical trials, to fully realize their translational potential and establish their efficacy in alleviating the burden of these debilitating conditions.

### 1.6. Molecular Structures of Key Bioactive Compounds

**Figure 2:**
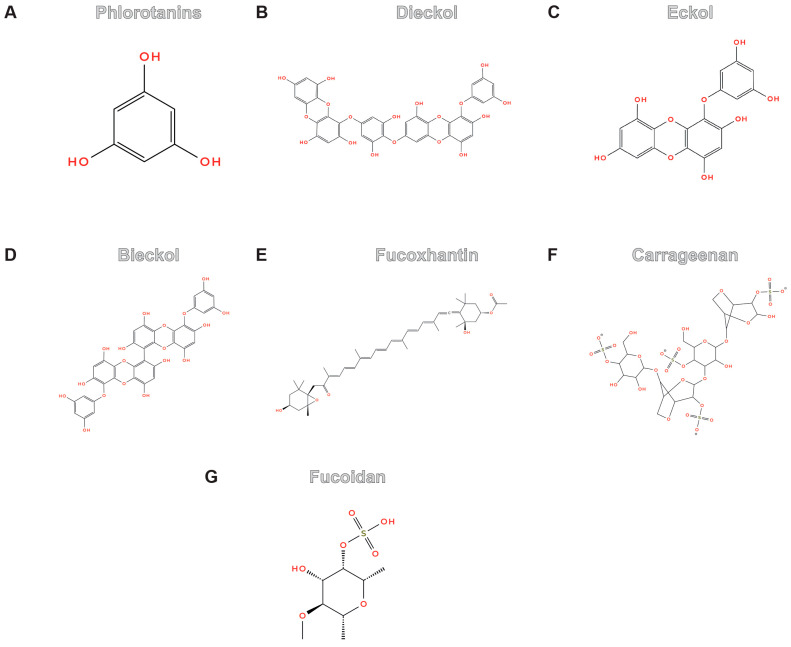
Molecular structures of key bioactive compounds. The molecular structures of the bioactive compounds investigated in this review are depicted as sketcher images. These structures were obtained and visualized using PubChem and Mol* Viewer (v4.10.0), accessed via a private subscription.

A clear depiction of the main structures of bioactive compounds is essential for understanding their inherent chemical characteristics, including stereochemistry and functional groups, which directly influence their biological activity and pharmacological profiles. The following visual representation aids in elucidating potential structure–activity relationships and provides a foundation for future drug design and optimization efforts (Figure 2).

## 2. Therapeutic Application in Pain Management: A Mechanistic Perspective

Marine algae represent a substantial reservoir of bioactive compounds with significant potential for pain reduction and modulation across a spectrum of pathological conditions. This potential is primarily attributable to their inherent antioxidant and anti-inflammatory activities. A considerable body of research, encompassing both preclinical and clinical investigations, has begun to elucidate the key mechanisms through which these marine organisms contribute to effective pain management. This potential is primarily attributed to their inherent antioxidant and anti-inflammatory activities. This manuscript has synthesized the current evidence and provides a rational argument for their therapeutic utility across various pain etiologies.

In the realm of inflammatory pain, research has demonstrated the efficacy of extracts derived from genera such *as Sargassum*, *Laminaria*, and *Ecklonia* in significantly downregulating the expression of pro-inflammatory cytokines, including IL-6 and TNF-α, in experimental models of rheumatoid arthritis. Notably, fucoidan, a sulfated polysaccharide present in brown algae, has been shown to inhibit the activation of NF-κB, a critical transcription factor in inflammatory processes. This inhibition results in a measurable reduction in joint inflammation and concomitant improvement in mobility [59,66,67]. Furthermore, marine polyphenols, such as phlorotannins isolated from *Fucus vesiculosus* and *Ascophyllum nodosum*, can suppress the production of inflammatory prostaglandins and nitric oxide (NO), thereby alleviating pain associated with chronic inflammatory conditions [65,68].

Neuropathic pain, characterized by aberrant neural signaling and hyperalgesia, has been investigated for various algae-derived compounds exhibiting promising effects. Fucoxanthin, a carotenoid present in *Undaria pinnatifida*, has been explored for its capacity to modulate microglial activation, a key driver of neuroinflammation in neuropathic pain. Preclinical studies suggest that this compound can attenuate the production of pro-inflammatory cytokines within the CNS, potentially leading to a reduction in hypersensitization associated with neuropathic pain [42,69]. Additionally, sulfated polysaccharides derived from *Chondrus crispus* and *Gracilaria lemaneiformis* have demonstrated regulatory effects on the transient receptor potential vanilloid 1 (TRPV1) receptor, which plays a crucial role in pain transmission. This suggests a potential analgesic effect in both peripheral and central neuropathies [70,71].

Muscle pain, whether resulting from fatigue or strenuous physical activity, represents another domain in which marine algae have demonstrated beneficial effects. Extracts from species such as *Porphyra umbilicalis* and *Ulva lactuca* exhibit significant antioxidant properties that can effectively mitigate exercise-induced oxidative stress. Research involving athletic populations has indicated that supplementation with these extracts leads to a reduction in the levels of malondialdehyde (MDA) and creatine kinase, established biomarkers of muscle damage, thereby facilitating acceleration and alleviating post-exercise muscle soreness [72,73]. Furthermore, these compounds have been observed to enhance mitochondrial function within muscle cells, optimizing energy production and reducing lactic acid accumulation, which can contribute to the prevention of muscle fatigue [74,75].

Bioactive compounds derived from marine algae have demonstrated promising effects in the context of chronic conditions such as fibromyalgia and multiple sclerosis. Sulfated polysaccharides from *Chondrus crispus* and *Kappaphycus alvarezii* have been investigated for their capacity to modulate systemic inflammation and regulate the expression of cyclooxygenase-2 (COX-2), a pivotal enzyme in inflammatory responses and pain perception [76,77]. Clinical trials involving patients with fibromyalgia have indicated that the administration of algal extracts can reduce pain hypersensitivity and enhance sleep quality, suggesting their potential as an adjunctive treatment strategy for managing this complex condition [78,79]. Furthermore, in experimental models of multiple sclerosis, marine polysaccharides have exhibited neuroprotective effects by attenuating microglial inflammation and promoting neuronal regeneration, which could have therapeutic implications for improving pain tolerance in these patients [80,81]. Finally, the efficacy of astaxanthin in mitigating hepatic damage induced by the microcystin-LR toxin has resulted in enhancements in liver health, attenuation of inflammation, and normalization of dysregulated lipid profiles [82]. Liver function, along with the inflammatory and oxidative mechanisms involved, is intricately associated with the pathophysiology of pain resulting from lipid metabolism disorders [83].

In conclusion, accumulating evidence strongly suggests that bioactive compounds derived from marine algae exhibit significant therapeutic potential in the management of inflammatory, neuropathic, muscular, and chronic pain. The elucidation of their diverse mechanisms of action, alongside their demonstrated efficacy in both preclinical and clinical studies, supports the potential integration of these compounds into pharmaceutical and nutraceutical formulations for natural pain management, potentially offering a favorable safety profile compared to conventional analgesic therapies. Nonetheless, it is crucial to acknowledge that further well-designed clinical trials are necessary to confirm their efficacy and safety in diverse human populations [42].

### Future Research Directions: Towards Etiopathogenesis-Driven Evaluation of Algal Analgesia

Current research highlights the importance of differential pain diagnosis, distinguishing between nociceptive pain, which arises from actual or threatened tissue damage, and neuropathic pain, which results from lesions or diseases of the somatosensory nervous system [84]. However, the potential influence of algal compounds on nociplastic pain, characterized by altered nociception despite no clear evidence of actual or threatened tissue damage or somatosensory lesions, remains unexplored. To advance our understanding, future investigations should prioritize a more granular approach by evaluating the analgesic efficacy of specific algal extracts or isolated compounds in the context of well-defined pain etiopathogenesis (Table 5). A prerequisite for such targeted research is a comprehensive understanding of the physiological processes modulated by various algal species, as summarized in previous sections. Furthermore, a rigorous examination of the etiology and origin of distinct pain types, coupled with a classification of the most pertinent physiological principles underlying their development, is crucial. We posit that each observed bioactivity of algal compounds may exert a distinct therapeutic mechanism contingent on the specific nature of the pain. Notably, certain compounds may demonstrate efficacy across multiple pain categories because of their capacity to modulate both inflammatory pathways and sensitization of the peripheral nervous system and CNS.

Therefore, future research in this domain should strategically prioritize evaluating the analgesic effects of individual algae or their bioactive constituents by explicitly considering the underlying etiopathogenesis of the pain condition under investigation. This refined approach promises to yield clinically translatable results, ultimately enhancing patient care. The overarching objective is not to propose an exclusive, singular treatment modality but rather to offer an evidence-based additional tool that can be integrated into a multimodal pain management strategy, potentially providing significant benefit to patients suffering from diverse pain conditions.

## 3. Conclusions

Marine algae constitute a rich and diverse source of bioactive compounds with considerable potential for therapeutic application in pain management. Compounds such as polyphenols, carotenoids, vitamins, minerals, and sulfated polysaccharides have exhibited antioxidant, anti-inflammatory, and neuroprotective properties, which are highly pertinent to the modulation of inflammatory and neuropathic pathways. Although much of the current evidence is derived from in vitro experiments and preclinical animal models, the findings are promising and suggest that these marine-derived compounds could serve as valuable adjuncts to conventional therapies. Notably, their natural origin and broad bioactivity profile may offer advantages in terms of safety and tolerability. Nonetheless, further well-designed clinical trials are essential to validate their efficacy, elucidate their mechanisms of action in human physiology, and ensure their safety across diverse populations. Overall, the expanding body of research supports the continued investigation of marine algae as a promising complementary approach for the development of novel, nature-based strategies for pain relief.

## Figures and Tables

**Table 1:** Quantification and mechanisms of action of polyphenols in algae.

Algae	Polyphenol	Quantification *	Mechanism of Action	Reference
*Fucus vesiculosus*	Phlorotannins	572.3 ± 3.2 mg/g DW	Inhibition of TNF-α and IL-6, reduction in inflammation and oxidative stress	[36]
*Ecklonia cava*	Dieckol, Eckol Bieckol	18.09 mg/g DW	Inhibition of COX-2 and NF-κB, reduction in pro-inflammatory cytokines	[37]
*Ascophyllum nodosum*	Phlorotannins,	143.1 mg/g DW 82.7 mg/g DW	Modulation of MMPs, cartilage protection in arthritis	[36]
*Laminaria japonica*	Phlorotannins	112.69 ± 2.85 mg/g DW	Reduction in oxidative stress, inhibition of IL-1β and PGE2	[38]
*Sargassum muticum*	Phlorotannins	94.0 mg/g DW	Regulation of the NF-κB pathway, reduction in inflammatory mediators	[36]
*Undaria pinnatifida*	Phlorotannins	10.7 ± 0.2 mg/g DW	Inhibition of ROS and modulation of the inflammatory response	[39]

Note: * mg gallic acid equivalent/g dry weight (GAE/g DW).

**Table 3:** Quantification and mechanisms of action of vitamins and minerals in algae.

Algae	Nutrient	Quantification	Mechanisms of Action	Reference
*Porphyra umbilicalis*	Vitamin C	1.61 mg/g DW	- Antioxidant: neutralizes ROS and protects cells from oxidative damage - Enhances immune function and collagen production	[55]
	Selenium	Present (not quantified)	- Antioxidant: component of enzymes that reduce cellular damage - Regulates immune function and inflammation	
*Ulva lactuca*	Vitamin E	0.0096–0.028 mg/g DW	- Protects cell membranes from oxidative damage - Reduces inflammation and improves cellular response to stress	[56,57,58]
*Gracilaria vermiculophylla*	Magnesium	Present (not quantified)	- Modulates NMDA receptor activity, reducing neuropathic pain - Relaxes muscles and improves nerve function	[55]
	Zinc	Present (not quantified)	- Regulates immune function and inflammation - Participates in cellular repair and antioxidant enzyme activity	

**Table 5:** Mechanisms of action of natural compounds in different types of pain.

Pain	Mechanism of Action	Explanation
*Nociceptive*	- Regulation of pro-inflammatory cytokines (TNF-α, IL-6, etc.) - Florotannins (such as polyphenols) - Inhibition of COX-2 expression and PGE2	Reduce oxidative stress and decrease the production of inflammatory mediators
*Neuropathic*	- Modulation of NMDA receptors - Reduction in microglial activation - Decreased production of ROS	Mitigate abnormal neuronal excitability, suppress microglial activation, and attenuate neuroinflammation
*Nociplastic*	- Oxidative stress and microglial activation - Regulation of the expression of genes involved in the immune response - Modulation of TRPV1 receptors	Modulate these pathways, including the regulation of pro-inflammatory genes in the CNS and the TRPV1 receptors, which are involved in pain perception

## Data Availability

The data presented in this study will be made available on request from the corresponding author.

## Abbreviations

The following abbreviations are used in this manuscript:

CNS	Central nervous system
COX-2	Cyclooxygenase-2
DW	Dry weight
GABA	GABAergic system
GAE	Gallic acid equivalent
IL-1β	Interleukin-1 beta
IL-6	Interleukin-6
MDA	Malondialdehyde
MMPs	Matrix metalloproteinases
NF-κB	Nuclear factor kappa-light-chain-enhancer of activated B cells
NMDA	N-Methyl-D-Aspartate
NO	Nitric oxide
PGE2	Prostaglandin E2
ROS	Reactive oxygen species
TNF-α	Tumor necrosis factor alpha
TRPV1	Transient receptor potential vanilloid 1
WHO	World Health Organization
